# *PCDHGB7* hypermethylation-based Cervical cancer Methylation (CerMe) detection for the triage of high-risk human papillomavirus-positive women: a prospective cohort study

**DOI:** 10.1186/s12916-024-03267-5

**Published:** 2024-02-05

**Authors:** Dan Cao, Zhicong Yang, Shihua Dong, Yuhong Li, Zhanrui Mao, Qi Lu, Peng Xu, Minfang Shao, Lei Pan, Xu Han, Jiangjing Yuan, Qiong Fan, Lei Chen, Yanzhong Wang, Weipei Zhu, Wenqiang Yu, Yudong Wang

**Affiliations:** 1 Department of Gynecology, The International Peace Maternity and Child Health Hospital, School of Medicine, Shanghai Jiaotong University, Shanghai Key Laboratory of Embryo Original Diseases, Shanghai, China; 2 Shanghai Public Health Clinical Center and Department of General Surgery, Huashan Hospital, Institutes of Biomedical Sciences, Shanghai Medical College, Fudan University, Shanghai, China; 3 Department of Obstetrics and Gynecology, Jinshan Hospital of Fudan University, Shanghai, China; 4 Department of Obstetrics and Gynecology, 2nd Affiliated Hospital of Soochow University, Suzhou, China; 5 Department of Population Health Sciences, School of Life Course and Population Sciences, King’s College London, London, UK

**Keywords:** CerMe detection, High-risk human papillomavirus, Triage

## Abstract

**Background:**

Implementation of high-risk human papillomavirus (hrHPV) screening has greatly reduced the incidence and mortality of cervical cancer. However, a triage strategy that is effective, noninvasive, and independent from the subjective interpretation of pathologists is urgently required to decrease unnecessary colposcopy referrals in hrHPV-positive women.

**Methods:**

A total of 3251 hrHPV-positive women aged 30–82 years (median = 41 years) from International Peace Maternity and Child Health Hospital were included in the training set (*n* = 2116) and the validation set (*n* = 1135) to establish Cervical cancer Methylation (CerMe) detection. The performance of CerMe as a triage for hrHPV-positive women was evaluated.

**Results:**

CerMe detection efficiently distinguished cervical intraepithelial neoplasia grade 2 or worse (CIN2 +) from cervical intraepithelial neoplasia grade 1 or normal (CIN1 −) women with excellent sensitivity of 82.4% (95% CI = 72.6 ~ 89.8%) and specificity of 91.1% (95% CI = 89.2 ~ 92.7%). Importantly, CerMe showed improved specificity (92.1% vs. 74.9%) in other 12 hrHPV type-positive women as well as superior sensitivity (80.8% vs. 61.5%) and specificity (88.9% vs. 75.3%) in HPV16/18 type-positive women compared with cytology testing. CerMe performed well in the triage of hrHPV-positive women with ASC-US (sensitivity = 74.4%, specificity = 87.5%) or LSIL cytology (sensitivity = 84.4%, specificity = 83.9%).

**Conclusions:**

*PCDHGB7* hypermethylation-based CerMe detection can be used as a triage strategy for hrHPV-positive women to reduce unnecessary over-referrals.

**Trial registration:**

ChiCTR2100048972. Registered on 19 July 2021.

**Supplementary Information:**

The online version contains supplementary material available at 10.1186/s12916-024-03267-5.

## Background

Cervical cancer is one of the leading causes of mortality among women worldwide [1]. As the first cancer type declared for elimination by 2030, screening and treatment of cervical intraepithelial neoplasia (CIN) greatly reduce mortality [2, 3]. The human papillomavirus (HPV) testing is the most widely used primary screening method with high sensitivity and extremely high negative predictive value (NPV) [4, 5]. However, most HPV infections are transient, and suboptimal specificity of the HPV testing results in unnecessary colposcopy referrals [6–8]. This drawback can be compensated by adequate triage strategies.

According to the current guidelines for cervical cancer prevention after HPV-based primary screening, hrHPV-positive patients are triaged by HPV genotyping or cytology [4, 5, 9, 10]. Women with positive HPV 16 and/or 18 are recommended to refer to colposcopy due to its correspondingly high risk for CIN grade 2 or worse (CIN2 + , including CIN2, CIN3, and cancer) [4, 10, 11]. However, over 90% of HPV16/18 infections will be cleared spontaneously after 2 years [12]. HPV genotyping-based triage test still leads to over-referrals and related side effects. Cytology testing has low sensitivity, unsatisfactory NPV, and poor reproducibility, and interpretation by pathologists is affected by subjective factors [13, 14]. Atypical squamous cells of undetermined significance (ASC-US) are the most common cytological abnormality. Reports indicate that a diagnosis of CIN 3 is made in only 7–10% of ASC-US cases and diagnoses of invasive carcinoma in women with ASC-US are rare [15]. Cytology testing with ≥ ASC-US cytology as a triage condition also increases unnecessary referrals. Therefore, an effective, noninvasive, and objective triage method is urgently needed for hrHPV-positive women to decrease unnecessary referrals.

Novel technologies for the detection of cervical cancer and precancerous lesions have been developed and evaluated, such as immunohistochemistry [16], microRNA test [17], and testing for E6/E7 or other oncogene aberrations [18, 19] and virus DNA integration [20]. Although most of these tools have suboptimal performance or high technical requirements, p16 still received considerable research attention. p16/Ki-67 double staining known as CINtec Plus Cytology can also be used as an indicator for long-term risk stratification to identify transforming HPV infection, and its NPV was superior to normal cytological results [21]. It is recommended that an HSIL cytology result and/or dual p16/Ki-67 staining could be the best candidates for colposcopy in HPV-positive patients [22]. However, this tool still relies heavily on the experience of pathologists, and further randomized studies are required before routine practice [23].

DNA methylation abnormalities have been shown to precede pathological changes [24, 25]. Besides, molecular testing of DNA methylation is objective, independent of professional pathologists, and automated approaches are anticipated, conferring a unique advantage over other various detection methods [26]. DNA methylation aberrations of specific genes including *miR124-2*, *FAM19A4*, *ASTN1*, *DLX1*, *ITG4*, *RXFP3*, *SOX17*, *ZNF671*, *TERT*, and other frequent methylation sites are associated with CIN and cervical cancer [27]. However, unsatisfactory analytical performance [28], complicated model construction process [29], and/or methodological limitations [30] have largely impeded the translation of the initial discovery into a clinical test. A systematic review summarizing 43 studies with 16,336 women included *CADM1*, *MAL*, *MIR-124–2*, *FAMI19A4*, *POU4F3*, *EPB41L3*, *PAX1*, *SOX1*, and HPV16 (L1/L2) methylation, and only a few markers achieved optimal sensitivity and/or specificity [31].

Our previous research identified hypermethylated *PCDHGB7* as a novel cancer marker with discernible value for early cervical cancer detection and modified methylation-sensitive restriction enzyme qPCR (MSRE-qPCR) to quantify its methylation status [32]. The combination of specific *PCDHGB7* hypermethylation site and bisulfite-free technique is more stable, convenient, quick, and cost-effective than conventional methylation detection methods. In the present study, we further optimized the evaluation system for methylation levels and proposed Cervical cancer Methylation (CerMe) detection. We applied this approach to a large and prospective screening cohort and investigated the diagnostic performance of CerMe detection in the triage of hrHPV-positive women.

## Methods

### Study design and participants

In this prospective and blinded study, all patients meeting all of the following inclusion criteria in the outpatient department of the International Peace Maternity and Child Health Hospital were recruited between August 1, 2021, and August 1, 2022: (1) aged ≥ 30 years, (2) undergo HPV and cytology testing in our institution and hrHPV-positive, and (3) agree to use the remaining HPV testing samples for this study. Firstly, we excluded patients missing cytology information, and the remaining cervical brush samples of HPV testing of the enrolled patients were collected with written informed consent. All of them were referred for colposcopy, and their remaining samples were blinded when being transferred to laboratory personnel for methylation detection. During this step, patients with one of the following criteria were excluded: (1) samples failed quality control (the volume of remaining samples was less than 400 μl) and (2) failed assay. Two months after the last patient was enrolled, the sample information was unblinded. Patients meeting any of the following criteria were excluded: (1) lost to follow-up without a colposcopy visit; (2) a diagnosis of other types of cancer, such as endometrial cancer and ovarian cancer; (3) vaginal or vulval intraepithelial neoplasia grade 2 or worse; and (4) a history of CIN2 + . Finally, the methylation results and clinical information of eligible patients were included in the analysis for the establishment of CerMe detection and performance evaluation of subgroup analyses. Institutional Review Board approval for research on human subjects was obtained from the Ethics Committee of International Peace Maternity and Child Health Hospital (License Number: GKLW-2020–22). This study has been registered on ClinicalTrials.gov (ChiCTR2100048972).

### CerMe detection

A methylation test was performed using the remaining cervical brush samples of HPV testing. A volume of 400 μl of remaining samples of HPV testing was used for genomic DNA extraction by EP Genomic DNA Kit (Epiprobe Biotech, K-21) with an automated nucleic acid extraction instrument. Subsequently, 100 ng of genomic DNA was used for methylation-sensitive restriction enzyme qPCR (MSRE-qPCR) detection as described previously. Different from bisulfite PCR relying on bisulfite conversion, MSRE-qPCR is based on the selective digestion of DNA by methylation-sensitive enzyme followed by qPCR with primers that surround the cutting site [33]. We detected CpG sites for *PCDHGB7* genomic and *GAPDH* gene was used for normalization. The DNA methylation level for each sample was evaluated by ΔCt = Ct__*PCDHGB7*_ − Ct__*GAPDH*_, and ΔCt was further converted into CerMe value to assess the risk of cervical cancer, with higher CerMe values representing higher cervical cancer risk.

CerMe detection was performed by dedicated laboratory investigators who were masked to the results of cytology, HPV testing, and colposcopy until DNA methylation detection was completed. The diagnoses of doctors, HPV testers, and cell pathologists were independent of the CerMe detection. After specimens unblinding, participants were divided into a training set and validation set based on the incidence of the disease (normal, 80.0%; CIN1, 13%; CIN2/3, 6%; cancer, 1%). In chronological order of recruitment, the first 65% of cases were included in the training set (*n* = 2116), while the next 35% were included in the validation set (*n* = 1135). Both the training set and validation set were completely independent. The overall diagnostic accuracy was reflected by the area under the ROC curve. The Youden index was used to determine the cutoff value, and a cutoff of 1.0 was chosen when the Youden index was maximized. Samples with CerMe value below 1.0 were classified as methylation negative, while samples with CerMe value above 1.0 were defined as methylation positive.

### HPV testing

Cervix brush samples were obtained by gynecologists for the HPV testing by Roche Cobas HPV real-time PCR assay (Roche, Cobas 4800) following the manufacturer’s instructions. If a test was positive for either HPV type 16 or 18, the sample was classified as HPV16/18 positive. If a sample was HPV16/18 negative but positive for any of the other 12 HPV types (31, 33, 35, 39, 45, 51, 52, 56, 58, 59, 66, and 68), the sample was classified as other 12 hrHPV positive. The cervical brush samples were collected by doctors with gynecological qualifications in our institution, and HPV testing was carried out by clinical laboratory physicians with PCR testing qualifications.

### Cytology testing

Cytological sampling was performed with broom-type cervical smears (Rovers Medical Devices, Cervex-Brush®), and specimens were collected and preserved in SurePath™ Preservative Fluid. The Thinprep® 2000 System was applied to programmatically manage the test, including slice production and reading. The Bethesda System standard 2001 was used for cytological classification, as follows: (1) no intraepithelial lesion or malignancy (NILM); (2) ASC-US; (3) atypical glandular cells (AGCs); (4) atypical squamous cells, cannot exclude high-grade squamous intraepithelial lesion (ASC-H); (5) low-grade squamous intraepithelial lesion (LSIL); (6) high-grade squamous intraepithelial lesion (HSIL); (7) squamous cell carcinoma (SCC); and (8) adenocarcinoma (AC). Among these categories, AGC was classified as ASC-US; SCC and AC were classified as cervical cancer. When the cytological result was ≥ ASC-US, the sample was assessed as positive. The cytology test was conducted by two cell pathologists with more than 5 years of experience after qualification. If the results of the two were different, a third cell pathologist assisted in the diagnosis.

### Colposcopy biopsy

All hrHPV-positive women underwent colposcopy within 2 months of enrollment. Colposcopies were performed by qualified colposcopy specialists, and biopsies depended on the colposcopy images after acetic acid and iodine reagent staining. Colposcopies strictly followed the quality control requirements of colposcopy in the American Society of Colposcopy and Cervical Pathology (ASCCP) [10] and Chinese Society for Colposcopy and Cervical Pathology (CSCCP) guidelines [34]. Patients who met all of the following lowest risk criteria could be classified as normal without undergoing a cervical biopsy: (1) a completely normal colposcopic impression (i.e., no aceto-whitening, metaplasia, or other visible abnormality), (2) a transformation zone (TZ) of type 1, (3) aged < 40 years, (4) < HSIL cytology, and (5) without HPV16/18 infection. For those not meeting the lowest risk criteria above, multiple biopsies were performed on 2–4 points targeting the aceto-white areas. Endocervical curettage (ECC) was a mandatory procedure for patients with type 3 TZ, HPV16/18 infection, cytological HSIL, or aged ≥ 40 years. Whether the others underwent ECC was determined by colposcopists based on the images. The histopathology results were assessed by pathologists and categorized as follows: (1) normal; (2) CIN1; (3) CIN2/3, including CIN2, CIN2-3, and CIN3; and (4) cervical cancer, including AC, SCC, and cervical sarcoma. CIN1 − included normal and CIN1, while CIN2 + included CIN2/3 and CC. The colposcopists were blinded to the results of methylation until the histologic outcomes were obtained.

### Methylation combined Cytology (MeCy)

Since both CerMe and cytology have stratification values, we investigated whether the combination of both approaches would achieve greater consistency with pathological results and proposed Methylation combined Cytology (MeCy). CerMe and cytology were stratified into 4 × 6 different combinations, and the MeCy results were defined as four levels according to the incidence of CIN2 + in each combination. Patients with the incidence of CIN2 + below 4% were classified as MeCy negative, 4 to 25% as MeCy weakly positive, 25 to 75% as MeCy moderately positive, and 75 to 100% as MeCy strongly positive.

### Statistical analysis

The GraphPad Prism 9 and Microsoft Excel software were used to perform statistical analysis. The ROC curve was constructed to quantify the diagnostic performance using the hybrid Wilson/Brown method. The two-tailed unpaired parametric test was used to compare the difference in CerMe value between CIN1 − and CIN2 + . A *P* value less than 0.05 was considered significant (*P < 0.05, **P < 0.01, ***P < 0.001, ****P < 0.0001). The sensitivity, specificity, positive predictive value (PPV), NPV, and accuracy were calculated based on 2 × 2 tables. Missing data were removed from the analyses.

## Results

### The establishment of CerMe detection as a triage for hrHPV-positive women

**Fig. 1:**
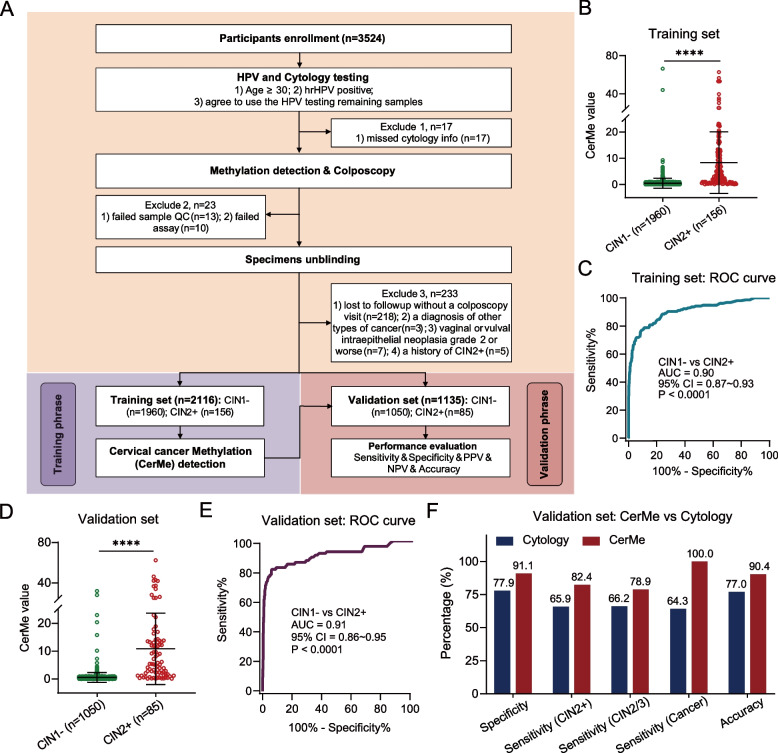
The establishment and verification of CerMe detection. **A** Flowchart of CerMe detection. CIN, cervical intraepithelial neoplasia; CIN1 − , including normal and CIN1; CIN2 + , including CIN2, CIN3, and cancer; HPV, human papillomavirus; hrHPV, high-risk human papillomavirus; NPV, negative predictive value; PPV, positive predictive value; QC, quality control. **B** CerMe values converted from the methylation level of *PCDHGB7* in the CIN1 − (*n* = 1960) and CIN2 + (*n* = 156) groups of the training set were shown in the scatter dot plot. The higher CerMe value corresponded to higher methylation status. **C** ROC curve and the associated AUC value of the CerMe detection in the training set are illustrated. **D** CerMe values of the CIN1 − (*n* = 1050) and CIN2 + (*n* = 85) groups in the validation set.** E** ROC curve and the associated AUC value of the CerMe detection in the validation set. **F** The specificity, sensitivity, and accuracy of cytology and CerMe detection in the validation set. In the scatter dot plot, the error bar represents mean ± SD. *P* values were calculated using the two-tailed unpaired parametric test by the GraphPad Prism 9 software. ****P < 0.0001

A total of 3251 hrHPV-positive women were enrolled and their HPV testing remaining samples were collected. The colposcopic biopsy result was used as the gold standard, and all of the patients underwent colposcopy and methylation tests. The age distribution of the included women is shown in Additional file 1: Fig. S1A. The distribution of the diagnoses of colposcopy biopsy and overall CerMe values is shown in the pie chart (Additional file 1: Fig. S1B) and bar chart (Additional file 1: Fig. S1C), respectively. Finally, 2116 samples were included in the training set (Additional file 1: Table S1). We analyzed the methylation status of specimens in the training set and established CerMe detection (Fig. 1A). Results showed that the CIN2 + group exhibited considerably higher CerMe values than CIN1 − (Fig. 1B). The area under the curve (AUC) of receiver operating characteristic (ROC) analysis in the training set was 0.90 (95% CI = 0.87 ~ 0.93) (Fig. 1C), and with a cutoff of CerMe value of 1.0, the specificity was 91.4% (95% CI = 90.1 ~ 92.6%), sensitivity was 76.3% (95% CI = 68.8 ~ 82.7%), and the overall accuracy rate was 90.3% (95% CI = 88.9 ~ 91.5%), which was superior to cytology (Additional file 1: Fig. S2). Further, 1135 cases were included in the subsequent verification. Detailed clinical characteristics of all included women are presented in Additional file 1: Table S1. Consistently, prominent higher level of CerMe values was also the case in CIN2 + women of the validation set (Fig. 1D) and AUC of ROC curve was 0.91 (95% CI = 0.86 ~ 0.95) (Fig. 1E). CerMe detection had higher sensitivity, specificity, and accuracy than cytology in distinguishing CIN2 + from CIN1 − patients (82.4% vs. 65.9%; 91.1% vs. 77.9%; 90.4% vs. 77.0%) (Fig. 1F and Additional file 1: Table S2). The sensitivity of CerMe detection in cervical cancer reached 100.0% (95% CI = 76.8 ~ 100.0%), while the sensitivity of cytology was only 64.3% (Fig. 1F). Collectively, the high consistency between the training set and validation set indicates the stability of CerMe detection.

We first evaluated the performance of CerMe detection and cytology testing by age stratification. Among hrHPV-positive women, CerMe outperformed cytology in the overall performance. Particularly, CerMe exhibited a high specificity of 94.6% in hrHPV-positive women aged 30–40 years, which was superior to cytology (76.8%). For hrHPV-positive women aged > 40 years, the sensitivity of CerMe reached 88.9% (cytology = 71.9%). The PPV of CerMe was significantly higher than that of cytology regardless of the age range of 30–40 years (46.0% vs. 15.1%) or older (39.7% vs. 19.4%), reducing the potential for overdiagnosis and overtreatment (Additional file 1: Fig. S3 and Table S3).

### CerMe detection as a triage for women infected with hrHPV of different subtypes

**Fig. 2:**
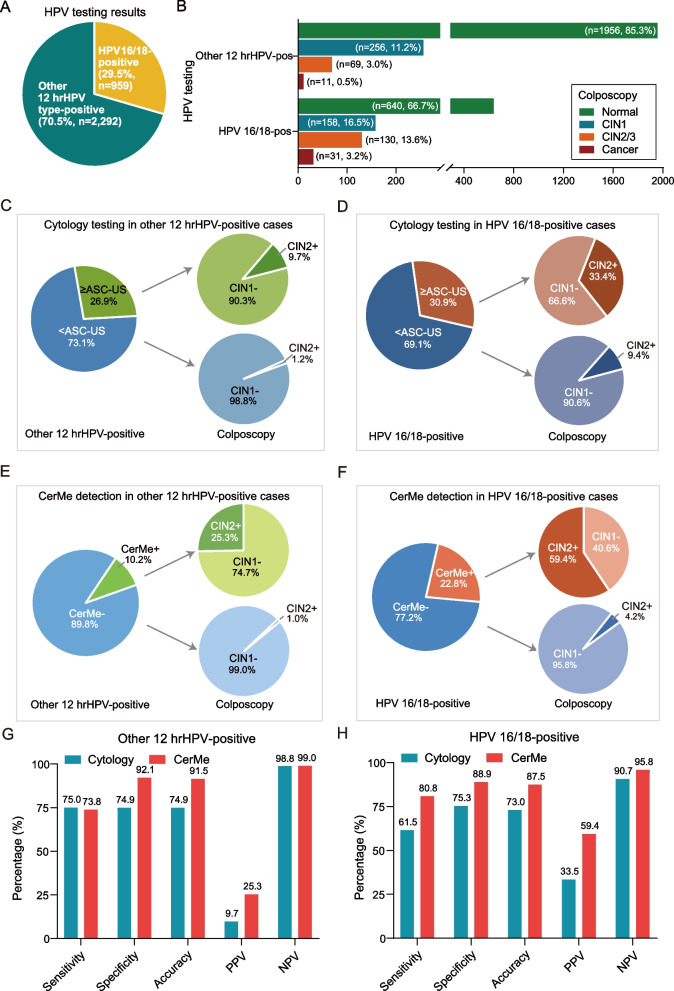
CerMe detection as a triage strategy for hrHPV-positive women. **A** The distribution of HPV16/18 and other 12 hrHPV types in hrHPV-positive women. **B** The proportion of colposcopic pathology in HPV16/18 and other 12 hrHPV type-positive women. **C** Cytology testing as a triage for other 12 hrHPV type-positive women.** D** Cytology testing as a triage for HPV16/18-positive women. **E** CerMe detection as a triage for other 12 hrHPV type-positive women. **F** CerMe detection as a triage for HPV16/18-positive women. **G** The performance of other 12 hrHPV type-positive women triaged by cytology testing or CerMe detection. **H** The performance of HPV16/18 type-positive women triaged by cytology testing or CerMe detection

A total of 161 of 959 (16.8%) HPV16/18-positive cases were finally diagnosed as CIN2 + by colposcopy, whereas the proportion of CIN2 + in the other 12 hrHPV type-positive cases was merely 3.5% (80/2292) (Fig. 2A, B), necessitating a triage strategy to efficiently identify patients with CIN2 + in this large population. There were 26.9% (616/2292) of the other 12 hrHPV type-positive and 30.9% (296/959) of HPV16/18-positive women with ≥ ASC-US cytology, and the proportions of CIN2 + in cases identified as < ASC-US cytology were 1.2% (20/1676) in other 12 hrHPV-positive cases and 9.4% (62/663) in HPV16/18-positive cases (Fig. 2C, D). By contrast, only 10.2% (233/2292) of the other 12 hrHPV type-positive and 22.8% (219/959) of HPV16/18-positive women were CerMe positive, and the proportions of CIN2 + in cases identified as CerMe negative were rare (other 12 hrHPV positive = 1.0%, 21/2059; HPV16/18 positive = 4.2%, 31/740) (Fig. 2E, F), indicating that methylation status may be considered as a practical risk indicator.

We compared the performance of CerMe detection with cytology testing as a triage protocol for hrHPV-positive women. Among the other 12 hrHPV-positive women, CerMe detection significantly reduced referrals by 62.2% (383/616) when compared with the triage of cytology (Additional file 1: Fig. S4A). Additionally, CerMe showed a similar sensitivity (73.8% vs. 75.0%), a superior specificity (92.1% vs. 74.9%), an improved accuracy (91.5% vs. 74.9%), and PPV (25.3% vs. 9.7%), as well as a nearly equivalent NPV (99.0% vs. 98.8%) compared to cytology (Fig. 2G and Additional file 1: Table S4). For HPV16/18 type-positive women, CerMe detection diminished referrals by 26.0% (77/296) compared to the triage of cytology (Additional file 1: Fig. S4B). CerMe exhibited a significantly higher sensitivity (80.8% vs. 61.5%), PPV (59.4% vs. 33.5%), specificity (88.9% vs. 75.3%), accuracy (87.5% vs. 73.0%), and NPV (95.8% vs. 90.7%) in comparison with cytology testing (Fig. 2H and Additional file 1: Table S4). Collectively, CerMe detection provided an optimal triage strategy for hrHPV-positive women to reduce over-referral for colposcopy, particularly in regions with strained medical resources.

### CerMe detection as a triage for hrHPV-positive women with ASC-US/LSIL cytology

**Fig. 3:**
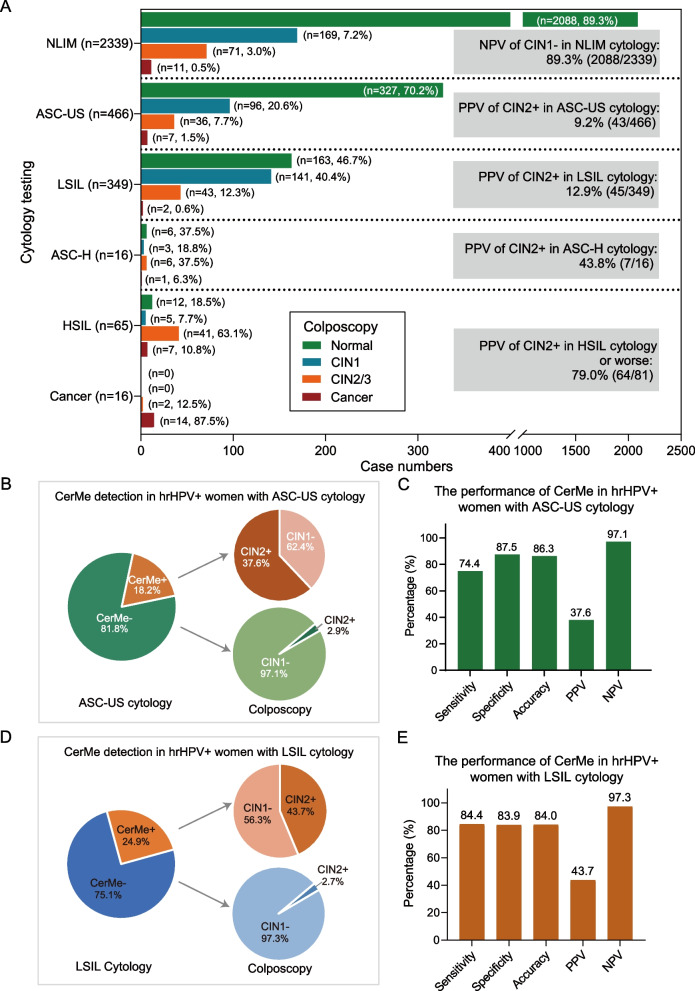
CerMe detection used for triage of hrHPV-positive women with ASC-US/LSIL cytology. **A** The proportion of pathology types on colposcopy in each of the six cytological diagnoses as well as the PPV of CIN2 + in cytological ASC-US, LSIL, ASC-H, HSIL, and cancer cases of the hrHPV-positive cohort. **B** CerMe detection as a triage for hrHPV-positive women with ASC-US cytology. **C** The performance of CerMe detection used as triage for hrHPV-positive women with ASC-US cytology. **D** CerMe detection as a triage for hrHPV-positive women with LSIL cytology. **E** The performance of CerMe detection used as triage for hrHPV-positive women with LSIL cytology. hrHPV + , hrHPV positive

Among hrHPV-positive women, 89.3% (2088/2339) of cases with NLIM cytology were diagnosed as CIN1 − by colposcopy (Fig. 3A). Despite ASC-US (14.3%, 466/3251) was the most frequent positive test result in this screening program followed by LSIL (10.7%, 349/3251) (Additional file 1: Fig. S5), only 9.2% (43/466) women with ASC-US cytology and 12.9% (45/349) women with LSIL cytology were diagnosed as CIN2 + by colposcopy, whereas women diagnosed as CIN2 + accounted for 73.9% (48/65) and 100% (16/16) of cases with HSIL and cancer cytology, respectively (Fig. 3A). There were many unnecessary referrals, particularly of hrHPV-positive women with ASC-US/LSIL cytology, and more definite detection is required to address this issue. Additionally, we observed that 81.8% (381/466) of cases with ASC-US cytology were methylation-negative. Of these, 97.1% (370/381) of cases were diagnosed as CIN1 − . Only 18.2% (85/466) of cases with ASC-US cytology were methylation positive. Of these, 37.6% (32/85) were finally diagnosed as CIN2 + (Fig. 3B). The sensitivity of CerMe detection triage for hrHPV-positive women with ASC-US cytology was 74.4% (95% CI = 58.8 ~ 86.5%), specificity was 87.5% (95% CI = 83.9 ~ 90.5%), and accuracy was 86.3% (95% CI = 82.8 ~ 89.3%) (Fig. 3C). Similarly, 75.1% (262/349) of cases with LSIL cytology were methylation-negative. Of these, 97.3% (255/262) of cases were diagnosed as CIN1 − . Only 24.9% (87/349) of cases with ASC-US cytology were methylation positive. Of these, 43.7% (38/87) were finally diagnosed as CIN2 + (Fig. 3D). The sensitivity of CerMe detection triage for hrHPV-positive women with LSIL cytology was 84.4% (70.5 ~ 93.5%), specificity was 83.9% (79.3 ~ 87.8%), and accuracy was 84.0% (79.7 ~ 87.7%) (Fig. 3E). Patients with ASC-US/LSIL cytology with a low-risk of CIN2 + could be effectively triaged by CerMe detection, while high-risk patients would be retained for continued attention, which greatly reduces the burden of referrals (Fig. 3B, D).

## CerMe stratification provides reference for colposcopic pathology

**Fig. 4:**
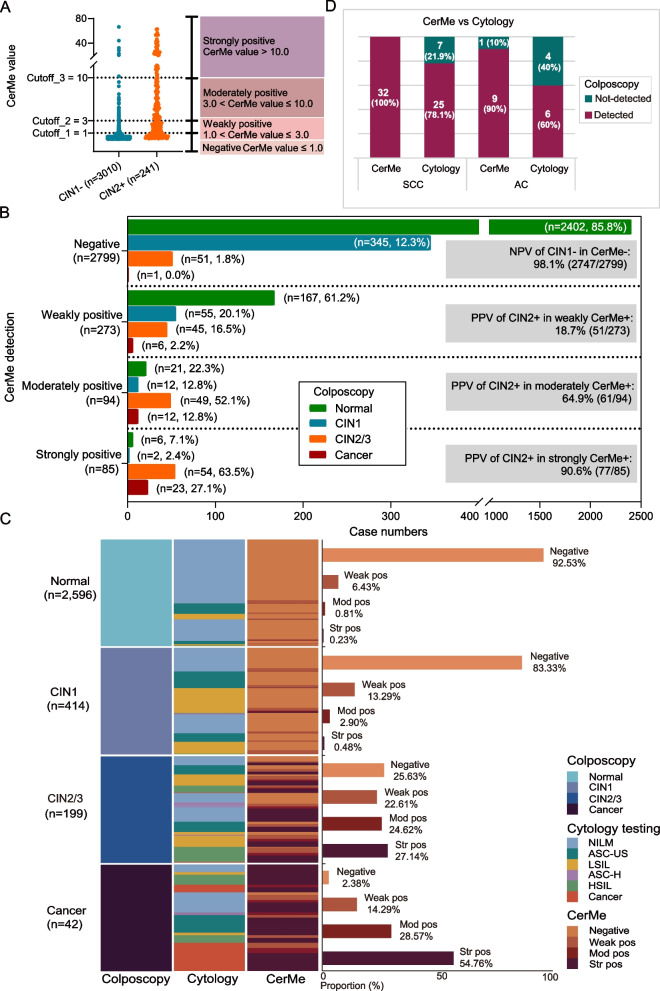
Stratified CerMe provided the reference for pathological diagnoses. **A** Stratification according to the CerMe values. **B** The proportion of pathology types on colposcopy in CerMe-positive (CerMe +) and CerMe-negative (CerMe −) cases as well as the PPV of CIN2 + in stratified CerMe + cases of the hrHPV-positive cohort. **C** Distributions of stratified CerMe (negative, weakly positive, moderately positive, and strongly positive) and cytology in colposcopic diagnoses. Bar chart showing the percentage of stratified CerMe cases in colposcopic diagnoses. **D** The sensitivity of CerMe and cytology testing in the detection of SCC and AC. SCC, squamous cell carcinoma; AC, adenocarcinoma

We hypothesized that the CerMe value reflects disease progression and further stratified the positive methylation results. A CerMe value between 1.0 and 3.0 with a PPV of 18.7% (51/273) was defined as weakly positive. A CerMe value between 3.0 and 10.0 with a PPV of 64.9% (61/94) was defined as moderately positive. A CerMe value greater than 10.0 was defined as strongly positive with a PPV of 90.6% (77/85) (Fig. 4A, B). Additionally, we compared the results of CerMe with cytological and colposcopic diagnoses. There were almost no methylation strongly positive cases in the groups categorized as normal (0.2%, 6/2596) and CIN1 (0.5%, 2/414) by colposcopy, while the percentage of strongly positive CerMe values in the groups categorized as CIN2/3 and cancer by colposcopy was 27.1% (54/199) and 54.8% (23/42), respectively (Fig. 4C). This suggested that patients with strongly positive CerMe deserved particular clinical attention.

According to the incidence of CIN2 + , cases were further divided into MeCy negative, MeCy weakly positive, MeCy moderately positive, and MeCy strongly positive. Results showed that the NPV of MeCy negative reached up to 98.2% (2726/2775) and the PPV of MeCy strongly positive (MeCy Str pos) was as high as 91.2% (114/125). The PPVs of MeCy moderately positive (MeCy Mod pos) and MeCy weakly positive were 54.5% (42/77) and 12.4% (34/274), respectively (Additional file 1: Fig. S6A). Especially, the group of MeCy strongly positive with PPV above 90% caught our attention. As the diagnostic performance of colposcopy strongly depends upon the subjective experience of operators [35], we conducted a re-analysis of outliers for 11 patients with MeCy strongly positive but negative colposcopic histopathology (normal or CIN1). Results of the second colposcopy biopsy showed that except for three patients lost to follow-up, the colposcopic pathology results were corrected from normal/CIN1 to CIN2/3 in 6 patients and corrected from normal to CIN1 in the remaining 2 patients (Additional file 1: Fig. S6B), indicating that methylation-positive stratification combined with cytology could provide a reference indicator for the risk assessment of missed diagnoses by colposcopy biopsy. Remarkably, the 9 outlier cases were all adenocarcinoma (AC). We compared the performance of CerMe with that of cytology in the detection of AC and SCC in hrHPV-positive women. Results showed that CerMe was more sensitive than cytology in the detection of both SCC (100% vs. 78.1%) and AC (90% vs. 60%) (Fig. 4D). This may to some extent compensate for the clinical diagnostic deficiencies of AC that are prone to a missed diagnosis.

## Discussion

In this study, we established CerMe, a *PCDHGB7* hypermethylation-based bisulfite-free detection, as a triage for hrHPV-positive women to reduce unnecessary over-referrals to colposcopy. We evaluated the performance of CerMe stratified by age, hrHPV subtypes, cytology, and CerMe values to prove its practicability.

Despite DNA methylation being heralded as a promising target for the development of cancer biomarkers, only a few markers have been successfully translated into clinical practice due to the complex detection methods and/or limited applicational performance. A test (Qiasure) detecting *FAM19A4*/*miR124-2* methylation showed a sensitivity of 68.0% and specificity of 78.3% for CIN2 + and 95.0% sensitivity for cervical cancer in a large multicenter cohort [36]. By contrast, CerMe detection showed superior performance with a sensitivity of 82.4% and a specificity of 91.1% for CIN2 + and 100% sensitivity for cervical cancer. Limited by the technical nature of bisulfate conversion, the invalid rate of *FAM19A4*/*miR124-2* methylation test reached 6% after undergoing optimization [36], while the invalid rate of CerMe detection was only 0.65% (23/3524) in this study, which enables its use for routine implementation. Furthermore, the clinical value of GynTect QSMP assay targeting *ASTN1*, *DLX1*, *ITG4*, *RXFP3*, *SOX17*, and *ZNF671* remains to be demonstrated [37]. Other epigenetic markers including *CADM1*, *MAL*, *EPB41L3*, *POU4F3*, *PAX1*, *JAM3*, *C13ORF18*, and *TERT* are under development [27, 38], while further clinical promotion requires proper detection techniques, such as next-generation sequencing, real-time quantitative methylation-specific polymerase chain reaction, or methylation microarrays. Bisulfite treatment, a necessary step in the above techniques, causes loss of material and genome complexity, reducing sensitivity in cancer detection [30]. Moreover, incomplete restriction digest and/or not all CpG sites in a given region are targetable by restriction enzymes in MSRE leading to a lack of specificity [33, 39]. Nevertheless, both targets and techniques were considered in CerMe detection to simultaneously portray both robustness and cost.

**Fig. 5:**
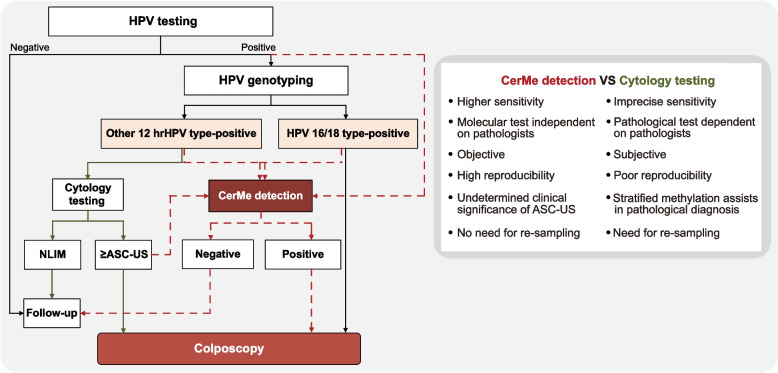
Protocol used to triage hrHPV-positive women in this study. The solid line represents the current protocol in clinical use, while the red dotted lines represent our recommended protocol

HPV-based screening could provide 60–70% greater protection against invasive cervical cancer compared with cytology testing [40]. However, recent studies suggested that for relatively young women, HPV testing and HPV16/18 genotyping may be suboptimal as it is difficult to determine whether the infection is transient or persistent [41, 42] and the actual risk for cervical cancer is hard to evaluate by qualitative results. We found that CerMe detection was associated with the degree of lesion, by which CIN1 − and CIN2 + , these two types of cervical lesions that face different cancer risks and clinical treatments, could be effectively discriminated, and quick action could be taken for women with an actual high risk. We also proposed a new protocol based on CerMe detection used for the triage of hrHPV-positive women (Fig. 5). The CerMe is a molecular test independent of the subjective experience of pathologists with a high sensitivity. The defined diagnostic criteria are objective, which decreases the interpretation error and eliminates the ambiguity of ASC-US. This triage strategy requires less training and the development of an automated system is anticipated. Also, re-sampling is not required, minimizing the burden on patients and physicians as well as limiting loss to follow-up.

Studies in adult women show younger patients had higher rates of regression and lower rates of progression [43, 44]. We found that CerMe outperformed cytology in specificity for hrHPV-positive women aged 30–40 years with high spontaneous regression rates, which avoids potential overdiagnosis and overtreatment. For hrHPV-positive women aged > 40 years with high progression rates, CerMe was superior to cytology in sensitivity, which avoids potential missed diagnoses. Our data support the superiority of CerMe detection. SCC is the most common histological type of cervical cancer. AC of the cervix, which develops from the glandular epithelium, accounted for 20–25% of cervical cancers in recent years [45]. AC is most likely to be located in the endocervical canal, which makes it more inconspicuous in cytology [46]. The rising prevalence and poor prognosis of AC pose considerable challenges for population screening programs [47–49]. In this study, CerMe detection was more sensitive than cytology for both SCC and AC detection, suggesting that it can be utilized as a complementary tool to cytology and HPV testing.

In conjunction with primary screening and final treatment, colposcopy plays an important role in guiding the follow-up process [50]. However, the accuracy of colposcopy is largely dependent on the subjective experience of colposcopists and pathologists [35]. The sensitivity of colposcopy for CIN2 + detection varies between 65 and 100% [51–53], and the misdiagnosis rate is higher in LMICs due to a shortage of experienced colposcopists. We consider that the finding that patients with a strongly positive CerMe had the highest incidence of CIN2 + with a PPV of 90.0% warrants particular clinical attention. Moreover, MeCy, combining stratified CerMe with cytology, could provide objective and quantitative reference indexes for colposcopists. Longitudinal studies with long-term follow-up are needed to validate the clinical feasibility of MeCy in assisting colposcopic pathology.

We described a group of > 3000 hrHPV-positive women who underwent cytology, colposcopy, and CerMe detection. To the best of our knowledge, this is the largest reported cohort and allows a systematic and comprehensive comparison of triage performance. Nonetheless, a limitation of this study is that it is based on a single screening independent of previous screening results; thus, participants included first-time screening and follow-up cases without a history of CIN2 + or other malignancies. Additionally, it is difficult to avoid missed diagnosis of colposcopy influenced by subjective factors. High-risk patients, particularly patients with a strongly positive CerMe, require further histological diagnosis involving loop electrosurgical excision procedure and endometrial biopsy to identify false-negative cases resulting from colposcopy.

These results demonstrated that CerMe detection served as a practicable triage approach for hrHPV-positive women to reduce unnecessary over-referrals and may provide a reference for pathological diagnosis. We intend to continue the follow-up of high-risk patients to clarify the predictive value of CerMe detection for disease progression.

## Conclusions

CerMe detection can be used as an effective, noninvasive, and objective triage strategy for hrHPV-positive women to reduce unnecessary over-referrals, which is an important supplement to the current screening guidelines for cervical cancer.

### Supplementary Information

**Additional file 1:  Fig. S1.** The clinical characteristics of the included women. (A) Histogram showing the age distribution of the included women. (B) Pie chart showing the percentage of patients diagnosed by colposcopy biopsy. (C) Bar chart showing the CerMe values’ distribution of the included women. **Fig. S2.** The specificity, sensitivity, and accuracy of cytology and CerMe detection in the training set. **Fig. S3.** The performance of cytology testing and CerMe detection stratified by age. (A) Pie chart showing the percentage of hrHPV-positive women aged 30-40 and >40 years. (B) The performance of cytology testing and CerMe detection in hrHPV-positive women aged 30-40 years. (C) The performance of cytology testing and CerMe detection in hrHPV-positive women aged >40 years. **Fig. S4.** CerMe detection as a triage protocol for hrHPV-positive women. (A) Flow of protocol used for other 12 hrHPV type-positive women. (B) Flow of protocol used for HPV 16/18 type-positive women. **Fig. S5.** The cytological characteristics of the included women. (A) Pie chart showing the percentage of patients diagnosed by cytology. (B) CerMe values of NLIM, ASC-US, LSIL, ASC-H, HSIL, and cancer cytology cases. **Fig. S6.** CerMe stratification combined with cytology (MeCy) provides reference for colposcopic pathology. (A) Heatmap of stratified CerMe combined Cytology (MeCy) showed the criteria of clustering MeCy negative, MeCy weakly positive, MeCy moderately positive, and MeCy strongly positive. Percentages on the 4 × 6 combinations showed the incidence of CIN2+. (B) Outlier analysis for 11 cases with strongly positive MeCy but negative colposcopy (normal or CIN1). Re-colposcopy, pathological results of a second colposcopy biopsy. NA, unavailable. **Table S1.** Clinical characteristics of all included women. **Table S2.** Diagnostic performance of hrHPV-positive women triaged by CerMe detection and Cytology testing. **Table S3.** Diagnostic performance of hrHPV-positive women aged 30-40 or >40 years. **Table S4.** Triage of other 12 hrHPV-positive or HPV 16/18-positive women.

## Data Availability

All data supporting the findings of this study are available within the main text or the additional files. Data sharing will be considered on reasonable request to the corresponding author.

## Abbreviations

AC: Adenocarcinoma
AGC: Atypical glandular cells
ASC-US: Atypical squamous cells of undetermined significance
ASC-H: Atypical squamous cells, cannot exclude high-grade squamous intraepithelial lesion
CerMe: Cervical cancer Methylation
CIN: Cervical intraepithelial neoplasia
hrHPV: High-risk human papillomavirus
HSIL: High-grade squamous intraepithelial lesion
LSIL: Low-grade squamous intraepithelial lesion
MeCy: Methylation combined Cytology
MSRE: Methylation-sensitive restriction enzyme
NILM: No intraepithelial lesion or malignancy
NPV: Negative predictive value
PPV: Positive predictive value
SCC: Squamous cell carcinoma
