# Supplementary material for: PCDHGB7 hypermethylation-based Cervical cancer Methylation (CerMe) detection for the triage of high-risk human papillomavirus-positive women: a prospective cohort study
Source: BMC Med. 2024 Feb 5;22:55. doi: 10.1186/s12916-024-03267-5 (PMC10845746; doi:10.1186/s12916-024-03267-5)
Supplement: Supplementary file 1 — Additional file 1: Fig. S1. The clinical characteristics of the included women. (A) Histogram showing the age distribution of the included women. (B) Pie chart showing the percentage of patients diagnosed by colposcopy biopsy. (C) Bar chart showing the CerMe values’ distribution of the included women. Fig. S2. The specificity, sensitivity, and accuracy of cytology and CerMe detection in the training set. Fig. S3. The performance of cytology testing and CerMe detection stratified by age. (A) Pie chart showing the percentage of hrHPV-positive women aged 30-40 and >40 years. (B) The performance of cytology testing and CerMe detection in hrHPV-positive women aged 30-40 years. (C) The performance of cytology testing and CerMe detection in hrHPV-positive women aged >40 years. Fig. S4. CerMe detection as a triage protocol for hrHPV-positive women. (A) Flow of protocol used for other 12 hrHPV type-positive women. (B) Flow of protocol used for HPV 16/18 type-positive women. Fig. S5. The cytological characteristics of the included women. (A) Pie chart showing the percentage of patients diagnosed by cytology. (B) CerMe values of NLIM, ASC-US, LSIL, ASC-H, HSIL, and cancer cytology cases. Fig. S6. CerMe stratification combined with cytology (MeCy) provides reference for colposcopic pathology. (A) Heatmap of stratified CerMe combined Cytology (MeCy) showed the criteria of clustering MeCy negative, MeCy weakly positive, MeCy moderately positive, and MeCy strongly positive. Percentages on the 4 × 6 combinations showed the incidence of CIN2+. (B) Outlier analysis for 11 cases with strongly positive MeCy but negative colposcopy (normal or CIN1). Re-colposcopy, pathological results of a second colposcopy biopsy. NA, unavailable. Table S1. Clinical characteristics of all included women. Table S2. Diagnostic performance of hrHPV-positive women triaged by CerMe detection and Cytology testing. Table S3. Diagnostic performance of hrHPV-positive women aged 30-40 or >40 years. Table S4. Tri [file 12916_2024_3267_MOESM1_ESM.docx]

**Additional file 1**


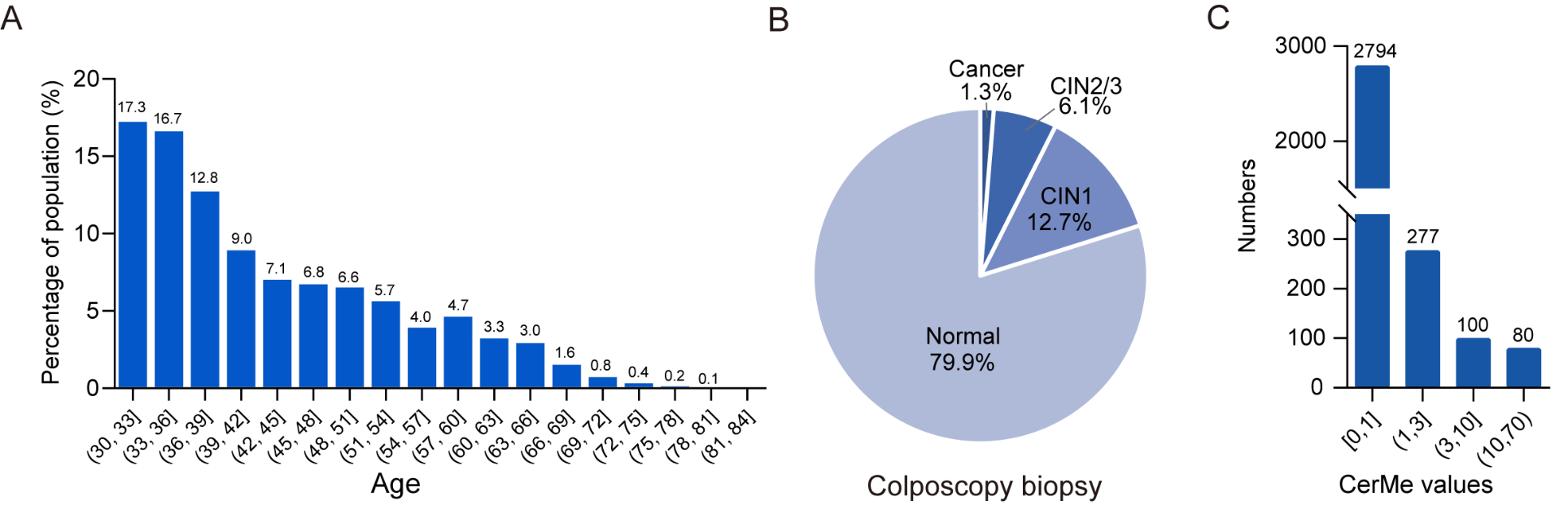


**Fig. S1** The clinical characteristics of the included women. (**A**) Histogram showing the age distribution of the included women. (**B**) Pie chart showing the percentage of patients diagnosed by colposcopy biopsy. (**C**) Bar chart showing the CerMe values’ distribution of the included women.


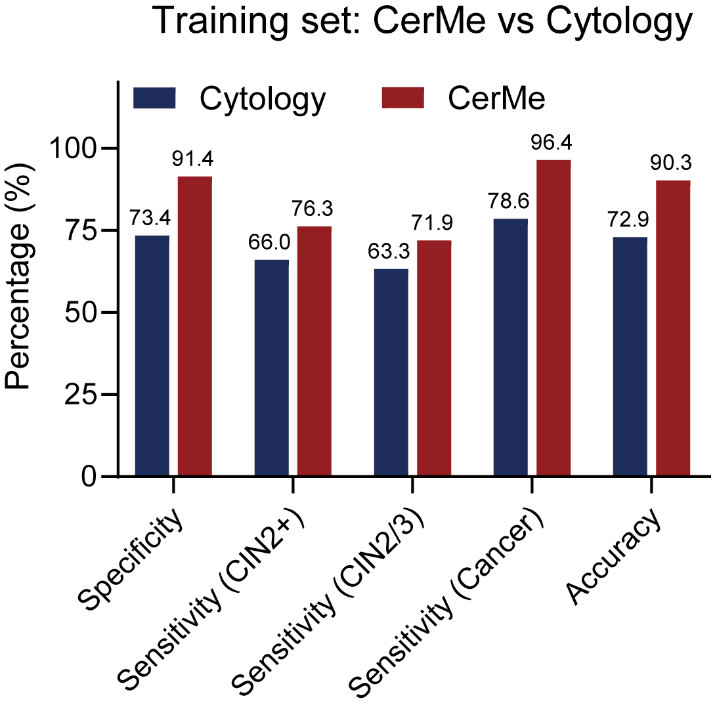


**Fig. S2** The specificity, sensitivity, and accuracy of cytology and CerMe detection in the training set.


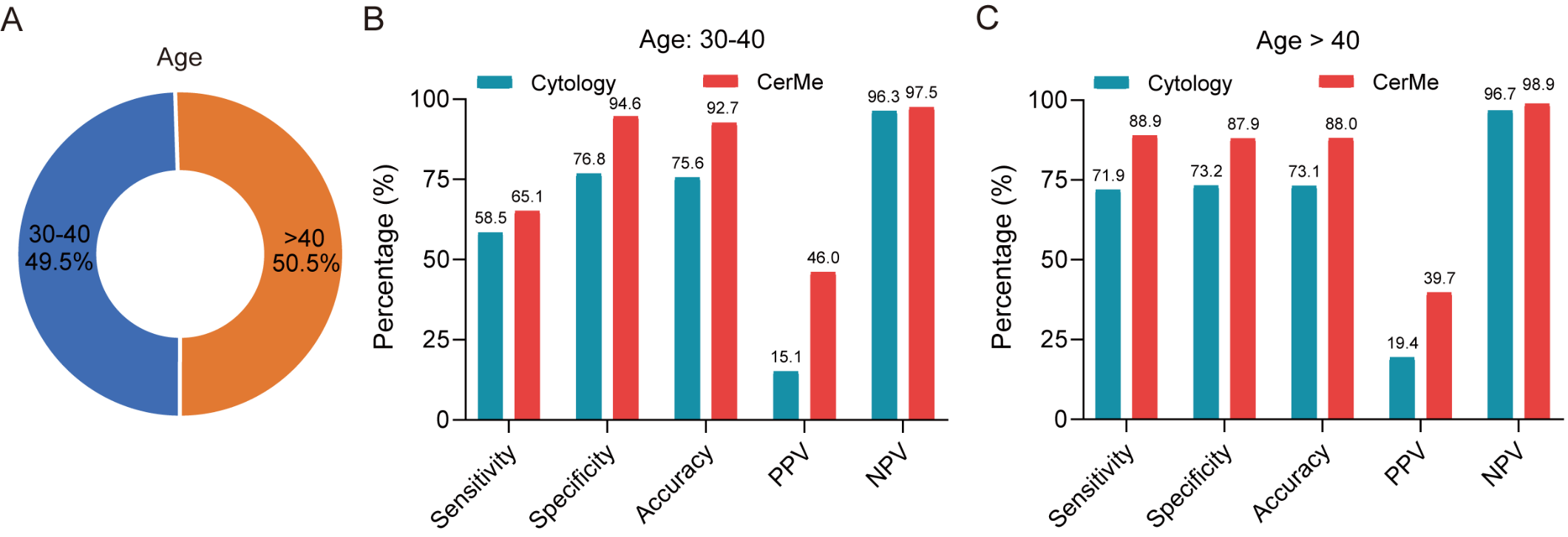


**Fig. S3** The performance of cytology testing and CerMe detection stratified by age. (**A**) Pie chart showing the percentage of hrHPV-positive women aged 30-40 and >40 years. (**B**) The performance of cytology testing and CerMe detection in hrHPV-positive women aged 30-40 years. (**C**) The performance of cytology testing and CerMe detection in hrHPV-positive women aged >40 years.


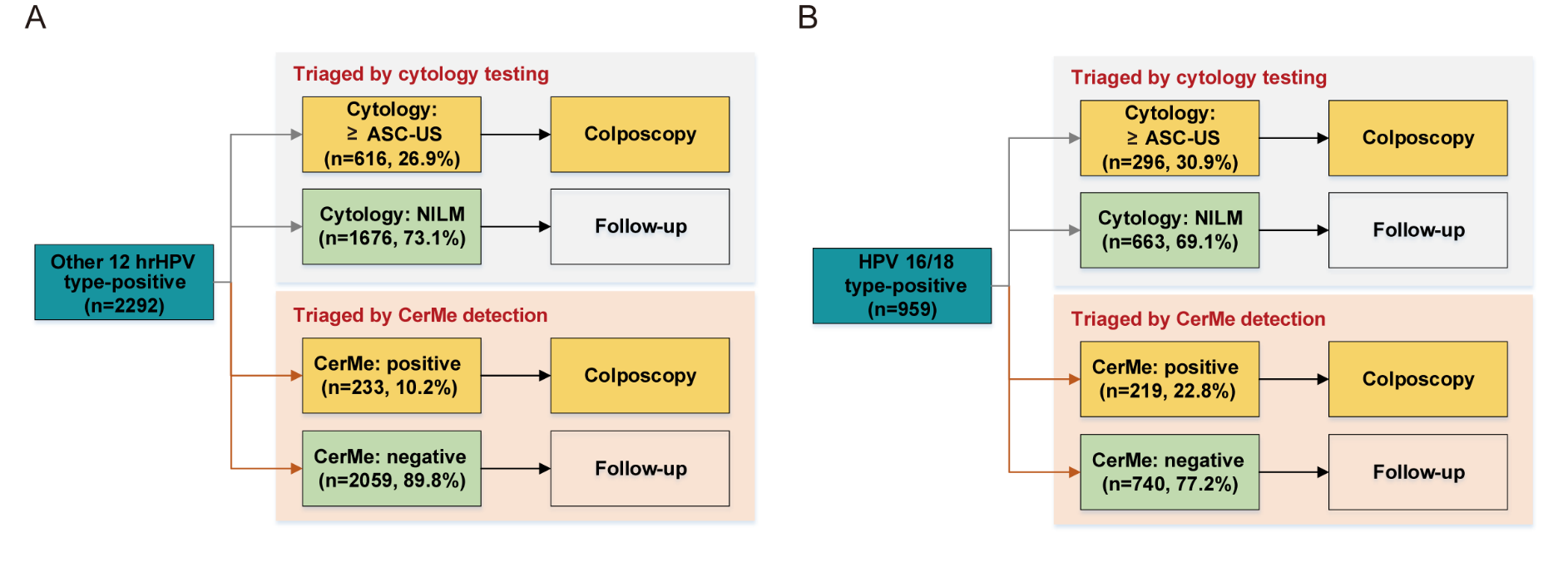


**Fig. S4** CerMe detection as a triage protocol for hrHPV-positive women. (**A**) Flow of protocol used for other 12 hrHPV type-positive women. (**B**) Flow of protocol used for HPV 16/18 type-positive women.


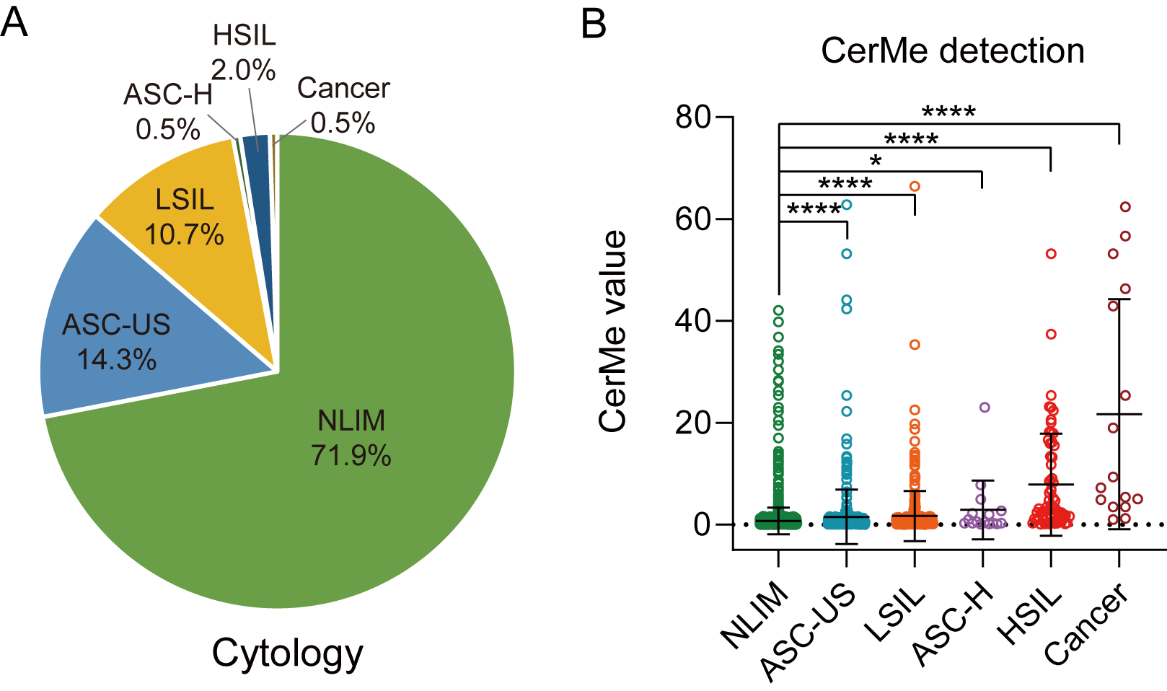


**Fig. S5** The cytological characteristics of the included women. (**A**) Pie chart showing the percentage of patients diagnosed by cytology. (**B**) CerMe values of NLIM, ASC-US, LSIL, ASC-H, HSIL, and cancer cytology cases.

**
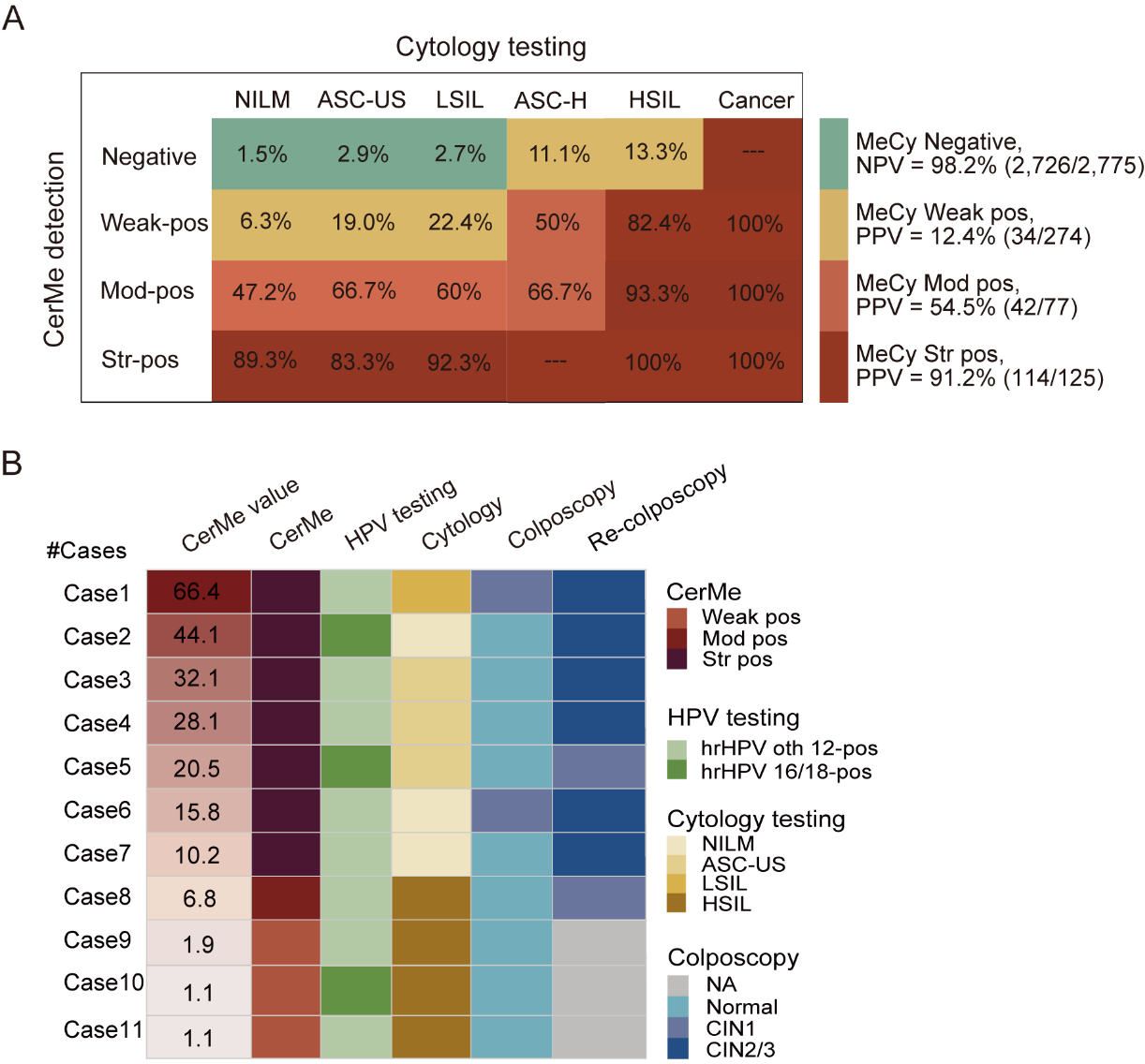
**

**Fig. S6** CerMe stratification combined with cytology (MeCy) provides reference for colposcopic pathology. (**A**) Heatmap of stratified CerMe combined Cytology (MeCy) showed the criteria of clustering MeCy negative, MeCy weakly positive, MeCy moderately positive, and MeCy strongly positive. Percentages on the 4 × 6 combinations showed the incidence of CIN2+. (**B**) Outlier analysis for 11 cases with strongly positive MeCy but negative colposcopy (normal or CIN1). Re-colposcopy, pathological results of a second colposcopy biopsy. NA, unavailable.

**Table S1** Clinical characteristics of all included women.

| **Characteristics** | **Training set (n=2116)** | **Validation set (n=1135)** |
| --- | --- | --- |
| **Age (year) (Range, median)** | 30~82, 41 | 30~77, 40 |
| **HPV testing results (n, %)** | | |
| HPV 16/18 type-positive | 589, 27.8% | 370, 32.6% |
| Other 12 hrHPV type-positive^*1^ | 1527, 72.2% | 765, 67.4% |
| **Cytology testing results (n, %)** | | |
| NILM | 1492, 70.5% | 847, 74.6% |
| ASC-US  ASC-H | 331, 15.6%  15, 0.7% | 135, 11.9%  1, 0.1% |
| LSIL | 222, 10.5% | 127, 11.2% |
| HSIL | 47, 2.2% | 18, 1.6% |
| Cervical cancer | 9, 0.4% | 7, 0.6% |
| **Colposcopy results (n, %)** | | |
| Normal | 1691, 79.9% | 905, 79.7% |
| CIN1 | 269, 12.7% | 145, 12.8% |
| CIN2/3 | 128, 6.1% | 71, 6.3% |
| Cervical adenocarcinoma | 6, 0.3% | 4, 0.4% |
| Cervical squamous carcinoma | 22, 1.0% | 10, 0.9% |
| **CerMe detection (n, %)** | | |
| Negative | 1828, 86.4% | 971, 85.6% |
| Weak positive | 178, 8.4% | 95, 8.4% |
| Middle positive | 64, 3.0% | 30, 2.6% |
| Strong positive | 46, 2.2% | 39, 3.4% |

Note: NILM, no intraepithelial lesion or malignancy; ASC-US, atypical squamous cells of undetermined significance; LSIL, low-grade squamous intraepithelial lesion; HSIL, high-grade squamous intraepithelial lesion; HPV, human papillomavirus. CIN, cervical intraepithelial neoplasia.

^*1^ other 12 hrHPV type includes 31, 33, 35, 39, 45, 51, 52, 56, 58, 59, 66, and 68.

**Table S2** Diagnostic performance of hrHPV-positive women triaged by CerMe detection and Cytology testing.

|  | Training set (n=2116) | | | |  | Validation set (n=1135) | | | |
| --- | --- | --- | --- | --- | --- | --- | --- | --- | --- |
|  | CerMe detection | | Cytology testing | |  | CerMe detection | | Cytology testing | |
|  | N/All | Percent, 95% CI (%) | N/All | Percent, 95% CI (%) |  | N/All | Percent, 95% CI (%) | N/All | Percent, 95% CI (%) |
| Specificity | 1791/1960 | 91.4, 90.1 - 92.6 | 1439/1960 | 73.4, 71.4 - 75.4 |  | 956/1050 | 91.1, 89.2 - 92.7 | 818/1050 | 77.9, 75.3 - 80.4 |
| Sensitivity (CIN2+) | 119/156 | 76.3, 68.8 - 82.7 | 103/156 | 66.0, 58.0 - 73.4 |  | 70/85 | 82.4, 72.6 - 89.8 | 56/85 | 65.9, 54.8 - 75.8 |
| Sensitivity (CIN2/3) | 92/128 | 71.9, 63.3 - 79.5 | 81/128 | 63.3, 54.3 - 71.6 |  | 56/71 | 78.9, 67.6 - 87.7 | 47/71 | 66.2, 54.0 - 77.0 |
| Sensitivity (Cancer) | 27/28 | 96.4, 81.7 - 99.9 | 22/28 | 78.6, 59.1 - 91.7 |  | 14/14 | 100.0, 76.8 - 100.0 | 9/14 | 64.3, 35.1 - 87.2 |
| PPV | 119/288 | 41.3, 35.6 - 47.2 | 103/624 | 16.5, 13.7 - 19.7 |  | 70/164 | 42.7, 35.0 - 50.6 | 56/288 | 19.4, 15.0 - 24.5 |
| NPV | 1791/1828 | 98.0, 97.2 - 98.6 | 1439/1492 | 96.4, 95.4 - 97.3 |  | 956/971 | 98.5, 97.5 - 99.1 | 818/847 | 96.6, 95.1 - 97.7 |
| Accuracy | 1910/2116 | 90.3, 88.9 - 91.5 | 1542/2116 | 72.9, 70.9 - 74.8 |  | 1026/1135 | 90.4, 88.5 - 92.1 | 874/1135 | 77.0, 74.4 - 79.4 |
| Sensitivity (SCC) | 22/22 | 100.0, 84.6 - 100.0 | 18/22 | 81.8, 59.7 - 94.8 |  | 10/10 | 100.0, 69.2 - 100.0 | 7/10 | 70.0, 34.8 - 93.3 |
| Sensitivity (AC) | 5/6 | 83.3, 35.9 - 99.6 | 4/6 | 66.7, 22.3 - 95.7 |  | 4/4 | 100.0, 39.8 - 100.0 | 2/4 | 50.0, 6.8 - 93.2 |

Note: AC, adenocarcinoma; CI, confidence interval; NPV, negative predictive value; PPV, positive predictive value; SCC, squamous cell carcinoma.

**Table S3** Diagnostic performance of hrHPV-positive women aged 30-40 or >40 years.

|  | | **Sensitivity** | **Specificity** | **PPV** | **NPV** | **Accuracy** |
| --- | --- | --- | --- | --- | --- | --- |
| **Aged 30-40 years** | | | | | |  |
| Cytology testing | N/All | 62/106 | 1153/1502 | 62/411 | 1153/1197 | 1215/1608 |
|  | Percent (95% CI) | 58.5 (48.5 - 68.0) | 76.8 (74.5 - 78.9) | 15.1 (11.8 - 18.9) | 96.3 (95.1 - 97.3) | 75.6 (73.4 - 77.6) |
| CerMe detection | N/All | 69/106 | 1421/1502 | 69/150 | 1421/1458 | 1490/1608 |
|  | Percent (95% CI) | 65.1 (55.2 -74.1) | 94.6 (93.3 - 95.7) | 46.0 (37.8 - 54.3) | 97.5 (96.5 - 98.2) | 92.7 (91.3 - 93.9) |
| **Aged >40 years** | | | | | |  |
| Cytology testing | N/All | 97/135 | 1104/1508 | 97/501 | 1104/1142 | 1201/1643 |
|  | Percent (95% CI) | 71.9 (63.5 -79.3) | 73.2 (70.9 - 75.4) | 19.4 (16.0 - 23.1) | 96.7 (95.5 - 97.6) | 73.1 (70.9 - 75.2) |
| CerMe detection | N/All | 120/135 | 1326/1508 | 120/302 | 1326/1341 | 1446/1643 |
|  | Percent (95% CI) | 88.9 (82.3 -93.7) | 87.9 (86.2 - 89.5) | 39.7 (34.2 - 45.5) | 98.9 (98.2 - 99.4) | 88.0 (86.3 - 89.5) |

Note: CIN2+, cervical intraepithelial neoplasia grade 2 or worse, including CIN2, CIN3, and cancer; CI, confidence interval; NPV, negative predictive value; PPV, positive predictive value.

**Table S4** Triage of other 12 hrHPV type-positive or HPV 16/18-positive women.

| **Triage strategies** | | **Sensitivity** | **Specificity** | **PPV** | **NPV** | **Accuracy** |
| --- | --- | --- | --- | --- | --- | --- |
| **Other 12 hrHPV type-positive**^*1^ | | | | | |  |
| Cytology testing | N/All | 60/80 | 1656/2212 | 60/616 | 1656/1676 | 1716/2292 |
|  | Percent (95% CI) | 75.0 (64.1 - 84.0) | 74.9 (73.0 - 76.7) | 9.7 (7.5 - 12.4) | 98.8 (98.2 - 99.3) | 74.9 (73.0 -76.6) |
| CerMe detection | N/All | 59/80 | 2038/2212 | 59/233 | 2038/2059 | 2097/2292 |
|  | Percent (95% CI) | 73.8 (62.7 - 83.0) | 92.1 (90.9 - 93.2) | 25.3 (19.9 - 31.4) | 99.0 (98.5 - 99.4) | 91.5 (90.3 -92.6) |
| **HPV 16/18-positive** | | | | | |  |
| Cytology testing | N/All | 99/161 | 601/798 | 99/296 | 601/663 | 700/959 |
|  | Percent (95% CI) | 61.5 (53.5 - 69.0) | 75.3 (72.2 -78.3) | 33.5 (28.1 -39.1) | 90.7 (88.2 -92.8) | 73.0 (70.1 -75.8) |
| CerMe detection | N/All | 130/161 | 709/798 | 130/219 | 709/740 | 839/959 |
|  | Percent (95% CI) | 80.8 (73.8 - 86.5) | 88.9 (86.5 - 91.0) | 59.4 (52.5 - 65.9) | 95.8 (94.1 - 97.1) | 87.5 (85.2 -89.5) |

Note: CIN2+, cervical intraepithelial neoplasia grade 2 or worse, including CIN2, CIN3, and cancer; CI, confidence interval; NPV, negative predictive value; PPV, positive predictive value.

^*1^ other 12 hrHPV type includes 31, 33, 35, 39, 45, 51, 52, 56, 58, 59, 66, and 68.
